# Improving Symbolic Automata Learning with Concolic Execution

**DOI:** 10.1007/978-3-030-45234-6_1

**Published:** 2020-03-13

**Authors:** Donato Clun, Phillip van Heerden, Antonio Filieri, Willem Visser

**Affiliations:** 8 University of Paderborn, Paderborn, Germany; 9 ICREA, Open University of Catalonia, Barcelona, Spain; 10 Imperial College London, London, UK; 11 Stellenbosch University, Stellenbosch, South Africa

## Abstract

Inferring the input grammar accepted by a program is central for a variety of software engineering problems, including parsers verification, grammar-based fuzzing, communication protocol inference, and documentation. Sound and complete active learning techniques have been developed for several classes of languages and the corresponding automaton representation, however there are outstanding challenges that are limiting their effective application to the inference of input grammars. We focus on active learning techniques based on $L^*$ and propose two extensions of the *Minimally Adequate Teacher* framework that allow the efficient learning of the input language of a program in the form of symbolic automata, leveraging the additional information that can extracted from concolic execution. Upon these extensions we develop two learning algorithms that reduce significantly the number of queries required to converge to the correct hypothesis.

### 1 Introduction

Inferring the input grammar of a program from its implementation is central for a variety of software engineering activities, including automated documentation, compiler analyses, and grammar-based fuzzing.

Several learning algorithms have been investigated for inferring a grammar from examples of accepted and rejected input words, with active learning approaches achieving the highest data-efficiency and strong convergence guarantees. Active learning is a theoretical framework enabling a *learner* to gather information about a target language by interacting with a *teacher* [1]. A minimally adequate teacher that can guarantee the convergence of an active language learning procedure for regular language is an oracle that can answer membership and equivalence queries. Membership queries check whether a word indicated by the learner is accepted by the target language and equivalence queries can confirm that a hypothesis language proposed by the learner is equivalent to the target language, or provide a counterexample word otherwise.

* This work has been partially supported by the EPSRC HiPEDS Centre for Doctoral Training (EP/L016796/1), the DSI-NRF Centre of Excellence in Mathematical and Statistical Sciences (CoE-MaSS), and a Royal Society Newton Mobility Grant (NMG\R2 \170142).

However, when learning the input language accepted by a program from its code implementation, it is unrealistic to assume the availability of a complete equivalence oracle, because such an oracle would need to check the equivalence between the hypothesis language and arbitrary software code.

In this paper, we explore the use of concolic execution to design active learning procedures for inferring the input grammar of a program in the form of a symbolic finite automaton. In particular, we extend two state of the art active learning frameworks for symbolic learning by enabling the teacher to 1) provide more informative answers for membership queries by pairing the accept/reject outcome with a path condition describing all the input words that would result in the same execution as the word indicated by the learner, and 2) provide a partial equivalence oracle that may produce counterexamples for the learner hypothesis. The partial equivalence oracle would rely on the exploration capabilities of the concolic execution engine to identify input words for which the acceptance outcome differs between the target program and the learner's hypothesis. To guarantee the termination of the concolic execution for equivalence queries, we set a bound on the length of the inputs the engine can generate during its exploration. While necessarily incomplete, such equivalence oracle may effectively guide the learning process and guarantee the correctness of the learned language for inputs up to the set input bound. Finally, we propose a new class of symbolic membership queries that build on the constraint solving capabilities of the concolic engine to directly infer complete information about the transitions between states of the hypothesis language.

In our preliminary evaluation based on Java implementations of parsers for regular languages from the Automatark benchmark suite, the new active learning algorithms enabled by concolic execution learned the correct input language for 76% of the subject, despite the lack of a complete equivalence oracle and achieving a reduction of up to 96% of the number of membership and equivalence queries produced by the learner.

The remaining of the paper is structured as follows: Section 2 introduces background concepts and definitions concerning symbolic finite state automata, active learning, and concolic execution. Section 3 describes in details the data structures and learning algorithms of two state of the art approaches – $\Lambda^*$ [11] and MAT* [3] – that will be the base for active learning strategies based on concolic execution formalized in Section 4. Section 5 will report on our preliminary experiments on the effectiveness and query-efficiency capabilities of the new strategies. Finally, Section 6 discusses related work and Section 7 presents our concluding remarks.

### 2 Preliminaries

#### 2.1 Symbolic finite state automata

Symbolic finite state automata (SFA) are an extension of finite state automata where a transitions can be labeled with a predicate identifying a subset of the input alphabet [28]. The set of predicates allowed on SFA transitions should constitute an *effective Boolean algebra* [3], which guarantees closure with respect to boolean operations according to the following definition:

**Definition 1.** *Effective Boolean algebra [3]. An effective Boolean algebra* $\mathcal{A}$ *is a tuple* $(\mathcal{D}, \Psi, [\![\_]\!], \bot, \top, \vee, \wedge, \neg)$ *where* $\mathcal{D}$ *is the set of domain elements;* $\Psi$ *is the set of predicates, including* $\bot$ *and* $\top$*;* $[\![\_]\!] : \Psi \to 2^{\mathcal{D}}$ *is a denotation function such that* $[\![\bot]\!] = \emptyset$*,* $[\![\top]\!] = \mathcal{D}$*, and for all* $\phi, \psi \in \Psi$*,* $[\![\phi \vee \psi]\!] = [\![\phi]\!] \cup [\![\psi]\!]$*,* $[\![\phi \wedge \psi]\!] = [\![\phi]\!] \cap [\![\psi]\!]$*, and* $[\![\neg\phi]\!] = \mathcal{D} \setminus [\![\phi]\!]$*.*

Given an effective Boolean algebra $\mathcal{A}$, an SFA is formally defined as:

**Definition 2.** *Symbolic Finite Automaton (SFA) [3]. A symbolic finite automaton* $\mathcal{M}$ *is a tuple* $(\mathcal{A}, Q, q_{init}, F, \Delta)$ *where* $\mathcal{A}$ *is an effective Boolean algebra, called the* alphabet*;* $Q$ *is a finite set of states;* $q_{init} \in Q$ *is the* initial state*;* $F \subseteq Q$ *is the set of* final states*; and* $\Delta \subseteq Q \times \Psi_{\mathcal{A}} \times Q$ *is the* transition relation *consisting of a finite set of* moves *or* transitions*.*

Given a linearly ordered finite alphabet $\Sigma$, through the rest of the paper we will assume $\mathcal{A}$ to be the Boolean algebra over the union of intervals over $\Sigma$, with the canonical interpretations of union, intersection, and negation operators. With an abuse of notation, we will write $\psi \in \mathcal{A}$ to refer to a predicate $\psi$ in the set $\Psi$ of $\mathcal{A}$. A *word* is a finite sequence of alphabet symbols (*characters*) $w = w_0 w_1 \dots w_n$ ($w_i \in \Sigma$), whose *length* $len(w) = n - 1$. We indicate with $w[: i]$ the prefix of $w$ up to the $i$ element excluded, and with $w[i :]$ the suffix of $w$ starting from element $i$. We will use the notation $w_i$ and $w[i]$ interchangeably. The language accepted by an SFA $\mathcal{M}$ will be indicated as $L_{\mathcal{M}}$, or only $L$ when the SFA M can be inferred by the context. For an SFA $\mathcal{M}$ and a word $w$, $\mathcal{M}(w) = true$ if $\mathcal{M}$ accepts $w$; $false$ otherwise.

Similarly to finite state automata, SFAs are closed under language intersection, union, and complement, and admit a minimal form [3]. Compared to non-symbolic automata, SFAs can produce more compact representations over large alphabets (e.g., Unicode), allowing a single transition predicate to account for a possibly large set of characters, instead of explicitly enumerating all of them.

#### 2.2 Active learning and minimally adequate teachers

Active learning encompasses a set of techniques enabling a learning algorithm to gather information interacting with a suitable oracle, referred to as *teacher*. Angluin [1] proposed an exact, active learning algorithm for a regular language $L$ named $L^*$. In $L^*$ the learner can ask the oracle two types of queries, namely *membership* and *equivalence* queries. In a membership query, the learner selects a word $w \in \Sigma^*$ and the oracle answers whether the $w \in L$ (formally, the membership oracle is a function $\mathcal{O}_m : \Sigma^* \to \mathbb{B}$, where $\mathbb{B} = \{true, false\}$). In an equivalence query, the learner selects an hypothesis finite state automaton (FSA) $\mathcal{H}$ and asks the oracle whether $L_{\mathcal{H}} \equiv L$; if $L_{\mathcal{H}} \not\equiv L$, the oracle returns a *counterexample*, i.e., a word $w$ in which $L$ differs from $L_H$ (formally, the equivalence oracle is a function $\mathcal{O}_e : FSA \to \Sigma^* \cup \{true\}$). A teacher providing both $\mathcal{O}_m$ and $\mathcal{O}_e$, and able to produce a counter example as result from $\mathcal{O}_e$ is called a *minimally adequate teacher*. Given a minimally adequate teacher, $L^*$ is guaranteed to learn the target language $L$ with a number of queries polynomial in the number of states of a minimal deterministic automaton accepting $L$ and in the size of the largest counterexample returned by the teacher [1].

**Discovering FSA states.** Consider an FSA $\mathcal{M}$. Given two words $u$ and $v$ such that $\mathcal{M}(u) \neq \mathcal{M}(v)$ (i.e., one accepted and one rejected), it can be concluded that $u$ and $v$ reach different states of $\mathcal{M}$. Moreover, if $u$ and $v$ share a suffix $s$ (i.e., $u = a.s$ and $v = b.s$ with $a, b, s \in \Sigma^*$ and the dot representing word concatenation), $a$ and $b$ necessarily reach two different states $q_a$ and $q_b$ of $\mathcal{M}$. The suffix $s$ is a *discriminator suffix* for the two states because its parsing starting from $q_a$ and $q_b$ leads to difference acceptance outcomes. The words $a$ and $b$ are instead *access words* of $q_a$ and $q_b$, respectively, because their parsing from the initial state reaches $q_a$ and $q_b$. This observation can be generalized to a set of words by considering all the unordered pairs of words in the set. Because $\mathcal{M}$ is a finite state automaton, there can be only a finite number of discriminable words in $\Sigma^*$ and, correspondingly, a finite number of distinct access string identifying the automaton's states.

**State reached parsing a word.** For a word $w$, consider a known discriminator suffix $s$ and access word $a$. If $\mathcal{O}_m(w.s) \neq \mathcal{O}_m(a.s)$, the state reached parsing $w$ cannot be the one identified by $a$. Throughout the learning process, it is possible that none of the already discovered access words identifies the state reached by $w$. In this case, $w$ would be a suitable candidate for discovering a new FSA state as described in the previous paragraph.

**Discovering FSA transitions.** For each access string $a$ and symbol $\sigma \in \Sigma$, a transition should exist between the states reached parsing $a$ and $a.\sigma$, respectively.

#### 2.3 Concolic execution

Concolic execution [14,27] combines concrete and symbolic execution of a program, allowing to extract for a given concrete input a set of constraints on the input space that uniquely characterize the corresponding execution path. To this end, the target program is instrumented to pair each program input with a symbolic input variable and to record along an execution path the constraints on the symbolic inputs induced by the encountered conditional branches. The conjunction of the constraints recorded during the execution of the instrumented program on a concrete input is called *path condition* and characterize the equivalence class of all the inputs that would follow the same execution path (in this paper, we focus on sequential program, whose execution is uniquely determined by the program inputs).

Explored path conditions can be stored in a prefix tree (*symbolic execution tree*), which captures all the paths already covered by at least one executed input. A concolic engine can traverse the symbolic execution tree to find branches not yet explored. The path condition corresponding to the selected unexplored branch is then solved using a constraint solver (e.g., an SMT solver [26]) generating a concrete input that will cover the branch. The traversal order used to find the next branch to be covered is referred to as *search strategy* of the concolic executor.

#### 2.4 From path conditions to SFA

In this paper, we consider only terminating programs that can either accept or reject a finite input word $w \in \Sigma^*$ (e.g., either parsing it correctly or throwing a parsing exception). Furthermore, we assume for a given input word $w$, the resulting path condition to be expressible using a subset of the string constraint language defined in [5]. This allows the translation of the resulting path condition into a finite state automaton [5]. The adaptation of this translation procedure to produce SFAs is straightforward. In particular, we will focus on constraints $F$ recursively defined as:

$$F \rightarrow C \mid \neg F \mid F \wedge F \mid F \vee F$$
$$C \rightarrow E\ O\ E \mid len(w)\ O\ E \mid w[n]\ O\ \sigma \mid w[len(w) - n]\ O\ \sigma$$
$$E \rightarrow n \mid n + n \mid n - n$$
$$O \rightarrow < \mid = \mid >$$

with $n \in \mathbb{Z}$ is an integer constant and $\sigma \in \Sigma$. Informally, the path condition corresponding to processing a symbolic input word $w$ should be reducible to a combination of interval constraints on the linearly ordered alphabet $\Sigma$ for each of the symbols $w[i]$ composing the input. Despite its restriction, this constraint language is expressive enough to capture the path conditions obtained from the concolic execution of a variety of programs that accept regular languages (which will be described in the evaluation section). The extension to support the entire string constraint language proposed in [5] is left as future work.

### 3 Active learning for SFA

Several active learning algorithms have been defined for SFAs. In this section, we recall and formalize the core routines of two extensions of $L^*$ proposed in [11] and [3], named $\Lambda^*$ and $MAT^*$, respectively. We will then extend and adapt these routines to improve their efficiency and resilience to incomplete oracles based on partial concolic execution.

**Running example.** To demonstrate the functioning of the algorithms discussed in this section and their extensions later one, we introduce here as running example the SFA accepting the language corresponding to regular expression `.*\w[^\w]\d[^\d].*`, where `\w` matches any letter, digit, or underscore (i.e., `[a-zA-Z0-9_]`), `\d` matches any digit, and `.*` matches any sequence of symbols. The regular expression is evaluated over the 16bit unicode symbols. The corresponding SFA is represented in Figure 1, where transitions are labeled by the union of disjoint intervals and each interval is represented as $\sigma_i - \sigma_j$, or $\sigma$ if it is composed by a single element; intervals are separated by a semicolon.

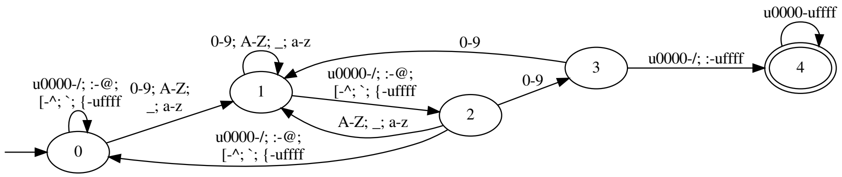

**Fig. 1.** SFA accepting the language for the running example.

This example highlights the conciseness of symbolic automata. It was chosen because the benefits of the methodologies discussed in this paper increase as the transitions are labeled with predicates representing larger set of characters, and the intervals used in this example are representative of commonly used ones.

#### 3.1 Learning using observation tables

$\Lambda^*$ is an adaptation of $L^*$ for learning SFAs. In both algorithms, the learner stores and process the information gathered by the oracle in an *observation table* (we adapt here the notation defined in [11]):

**Definition 3.** *Observation table [11]. An observation table $T$ for an SFA $\mathcal{M}$ is a tuple $(\Sigma, S, R, E, f)$ where $\Sigma$ is a potentially infinite set called the* alphabet*; $S, R, E \subset \Sigma^*$ are finite subsets of words called, respectively,* prefixes, boundary*, and* suffixes*. $f : (S \cup R) \times E \to \{true, false\}$ is a Boolean* classification function *such that for word $w \in (S \cup R)$ and $e \in E$, $f(w.e) = true$ iff $\mathcal{M}(w.e)$. Additionally, the following invariants hold: (i) $S \cap R = \emptyset$, (ii) $S \cup R$ is prefix-closed, and the empty word $\epsilon \in S$, (iii) for all $s \in S$, there exists a character $\sigma \in \Sigma$ such that $s.\sigma \in R$, and (iv) $\epsilon \in E$.*

Figure 2a shows an example observation table ($\mathbf{T}$) according to the notation in [11]. The rows are indexed by elements of $S \cup R$, with the elements of $S$ reported above the horizontal line and those of $R$ below it. The columns instead are indexed by elements of $E$. An element in $s \in S$ represent the access word to a state $q_s$, i.e., the state that would be reached by parsing $s$ from the initial state. Elements in the boundary set $R$ provide information about the SFA transitions. The elements of $e \in E$ are discrimination suffixes in that, if there exist $s_i, s_j \in S$ and $e \in E$ such that $f(s_i.e) \neq f(s_j.e)$, $s_i$ and $s_j$ reach different states of $\mathcal{M}$. The cell corresponding to a row index $w \in S \cup R$ and column index $e \in E$ contains the result of $f(w.e)$, which, for compactness, is represented as $+$ or $-$ when the $f$ evaluates to *true* or *false*, respectively. For an element $w \in S \cup R$, we use $row(w)$ to indicate the vector of $+/-$ in the row of the table indexed by $w$.

An observation table is: *closed* if for each $r \in R$ there exists $s \in S$ such that $row(r) = row(s)$; *reduced* if for all $s_i, s_j \in S$, $s_i \neq s_j \Rightarrow row(s_i) \neq row(s_j)$; *consistent* if for all $w_i, w_j \in S \cup R$ and $\sigma \in \Sigma$, if $w_i.\sigma, w_j.\sigma \in S \cup R$ and $row(w_i) = row(w_j)$ then $row(w_i.\sigma) = row(w_j.\sigma)$; *evidence-closed* if for all $e \in E$ and $s \in S$, $s.e \in S \cup R$. An observation table is *choesive* if it is closed, reduced, consistent, and evidence-closed. Informally, closed means that every element of $R$ corresponds to a state identified by an element of $S$; reduced, that every state is identified by a unique access string in $S$; consistent, that if two words $w_i$ and $w_j$ are equivalent according to $f$ and $E$, then also $w_i.\sigma$ and $w_j.\sigma$ should be equivalent for any symbol $\sigma \in \Sigma$.

**Induced SFA.** A cohesive observation table $\mathbf{T}$ induces an SFA that accepts or reject words consistently with its classification function $f$. Such induced SFA $\mathcal{M}_{\mathbf{T}} = (\mathcal{A}, Q, q_{init}, F, \Delta)$, where $\mathcal{A}$ is assumed to be the effective Boolean algebra over the union of intervals of $\Sigma$, is constructed as follows. For each $s \in S$ a corresponding state $q_s \in Q$ is defined, with the initial state $q_{init}$ being $q_\epsilon$. The final states $F$ are all the states $q_s$ such that $f(s) = true$. Since $\mathbf{T}$ is cohesive, a function $g : S \cup R \to S$ can be defined such that $g(w) = s$ iff $row(w) = row(s)$. Given $g$, for $w \in \Sigma^*$ and $\sigma \in \Sigma$, if $w.\sigma \in S \cup R$ then $(q_{g(w)}, \sigma, q_{g(w.\sigma)}) \in \Delta$. However, this intuitive construction of the transition relation $\Delta$ would result in

| $\mathbf{T}_0$ | $\epsilon$ |
|---|---|
| $\epsilon$ | - |
| A | - |

(a)

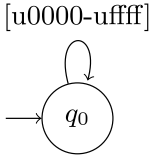

(b)

**Fig. 2.** Example of a cohesive observation table and its induced automata.

the construction of a FSA, where each transition is labeled with a single element $\sigma \in \Sigma$. To obtain an equivalent SFA, an additional step is required to learn the transition predicates of the SFA $\mathcal{M}_{\mathbf{T}}$.

**Transition predicates.** Given a Boolean algebra $\mathcal{A}$ with domain $\mathcal{D} = \Sigma$, a *partition function* can be defined that generalizes the concrete evidence for a transition of the induced automaton into a predicate of $\mathcal{A}$. Intuitively, the resulting predicate for a transition from state $q_i$ to state $q_j$ should evaluate positively for all the elements $\sigma_j \in \Sigma$ that would label a transition from $q_i$ to $q_j$ according to the function $g$ defined in the previous paragraph, and negatively for all the elements $\sigma_k$ that would label a transition from $q_i$ to a state other than $q_j$. Because the function $g$ is by construction a partial function (defined only for words $w.\sigma \in S \cup R$), the partition function can arbitrarily assign the symbols $\sigma$ not classified by $g$. This produces a natural generalization of the induced SFA from an observation table.

In this paper, we assume $\mathcal{A}$ to be the Boolean algebra over the union of intervals over $\Sigma$, with $\Sigma$ being a linearly ordered finite alphabet, such as the ascii or unicode symbols. For this algebra, a partition function can be trivially defined by constructing for each transition an interval union predicate characterizing all the concrete evidence symbols that would label the transition according to $g$. Then, for a given state, the symbols for which $g$ is not defined can be arbitrarily added to any of the predicates labeling an outgoing transition. A more efficient definition of a partition function for this algebra is beyond the scope of this section. The interested reader is instead referred to [11].

The introduction of a partition function to abstract concrete transition symbols into predicates of a Boolean algebra is the key generalization of $\Lambda^*$ over $L^*$ that allow learning SFAs instead of FSA. Going back to the observation table in Figure 2a, the induced SFA is shown in Figure 2b. The observation table provides concrete evidence for labeling the transition from $\epsilon$ to itself with the symbol A. The partition function generalized this concrete evidence into the predicate [u0000-uffff], which assigned all the elements of the unicode alphabet to the sole outgoing transition from $q_0$.

**Learning algorithm.** Initially, the learner assumes an observation table corresponding to the empty language, with $S = E = \{\epsilon\}$ and $R = \{\sigma\}$ for an arbitrary $\sigma \in \Sigma$, like the one in Figure 2a. The corresponding induced SFA $\mathcal{M}_{\mathbf{T}}$ is the hypothesis the learner proposes to the equivalence oracle $\mathcal{O}_e$. If the hypothesis does not correspond to the target language, the equivalence oracle returns a counterexample $c \in \Sigma^*$. There are two possible reasons for a counterexample: either a new state should be added to the current hypothesis or one of the predicates in the hypothesis SFA needs refinement. Both cases will be handled updating the observation table to include new evidence from the counterexample $c$, with the partition function automatically refining the transition predicates according to the new evidence in the table.

To update the observation table, first all the prefixes of $c$ (including $c$ itself) are added to $R$, except those already present in $S$. (We assume every time an element is added to $R$, the corresponding row is filled by issuing membership queries to determine the value of $f(r.e)$, $e \in E$, for each cell.) If for a word $r \in R$ there is no word $s \in S$ such that $row(r) = row(s)$, the word $r$ identifies a newly discovered state and it is therefore moved to $S$; a word $r.\sigma$ for an arbitrary $\sigma \in \Sigma$ is then added to $R$ to trigger the exploration of outgoing transitions from the newly discovered state. To ensure the updated observation table is evidence-closed, for all $s \in S$ and $e \in E$ $s.e$ and all its prefixes are added to $R$, if not already present. Finally, the observation table should be made consistent. To this end, if there exist and element $\sigma \in \Sigma$ such that $w_i, w_j, w_i.\sigma, w_j.\sigma \in S \cup R$ with $row(w_i) = row(w_j)$ but $row(w_i.\sigma) \neq row(w_j.\sigma)$, then $w_i$ and $w_j$ should lead to different states. Since $row(w_i.\sigma) \neq row(w_j.\sigma)$, there exist $e \in E$ such that $f(w_i.\sigma.e) \neq f(w_j.\sigma.e)$. Therefore, $a.e$ can discriminate between the states reached parsing $w_i$ and $w_j$ and as such $a.e$ should be added to $E$. The observation table is now cohesive and its induced SFA can be checked against the equivalence oracle, repeating this procedure until no counterexample can be found.

**Running example.** We demonstrate the first three iterations of the $\Lambda^*$ learning procedure invoked on the automaton in Figure 1. The initial table (Figure 2a) is cohesive, so an SFA is induced (Figure 2b) and an equivalence query is issued. The oracle returns the counter example $A!0B$. The counter example and its prefixes are added to the table (Figure 3a), and the table becomes open. The table is closed (Figure 3b), and becomes cohesive. An SFA is induced (Figure 3.1), and the equivalence query returns the counter example $B$. The counter example is added to the evidence (Figure 3c), and the table becomes consistent but open. The table is closed (Figure 3d), and becomes cohesive.

| $\mathbf{T}_1$ | $\epsilon$ |
|---|---|
| $\epsilon$ | - |
| A | - |
| A!0B | + |
| A!0 | - |
| A! | - |

(a) Add $A!0B$ to table.

| $\mathbf{T}_2$ | $\epsilon$ |
|---|---|
| $\epsilon$ | - |
| A!0B | + |
| A | - |
| A!0 | - |
| A! | - |

(b) Close.

| $\mathbf{T}_3$ | $\epsilon$ | B |
|---|---|---|
| $\epsilon$ | - | - |
| A!0B | + | + |
| A | - | - |
| A!0 | - | + |
| A! | - | - |
| B | - | - |

(c) Add $B$ to table and evidence.

| $\mathbf{T}_4$ | $\epsilon$ | B |
|---|---|---|
| $\epsilon$ | - | - |
| A!0B | + | + |
| A!0 | - | + |
| A | - | - |
| A! | - | - |
| B | - | - |

(d) Close.

**Fig. 3.** Observation tables for two iterations of $\Lambda^*$.

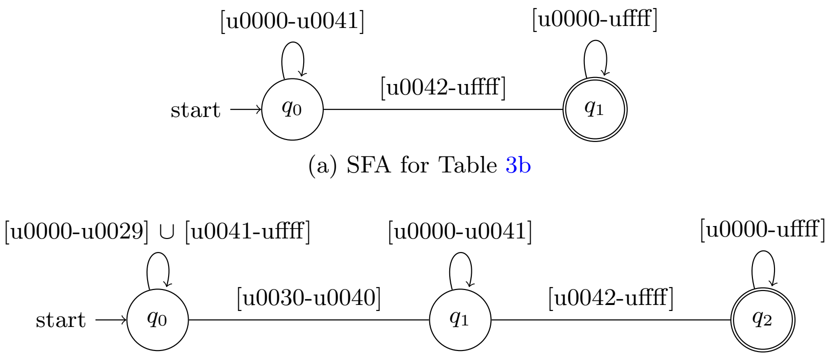

(a) SFA for Table 3b

(b) SFA for Table 3d

**Fig. 4.** Hypothesis automata for the learning iterations in Figure 3.

#### 3.2 Learning using discrimination trees

A discrimination tree (DT) is a binary classification tree used by the learner to store the information gathered from the teacher. Introduced in [23], it is the core data structure of several learning algorithms, including $TTT$ [20] and $MAT^*$ [3]. We formalize its structure and main routines that will be the baseline for extensions presented in the next section.

Recalling from Section 2.2, each state $q_a$ of an SFA $\mathcal{M}$ is identified by the learner using a unique *access word* $a \in \Sigma^*$. Given two states $q_a$ and $q_b$, $s \in \Sigma^*$ is a *discriminator suffix* for $q_a$ and $q_b$ if parsing $s$ starting from the two states leads to different outcomes (accept or reject). In terms of the state access words, this is equivalent to stating $\mathcal{M}(a.s) \neq \mathcal{M}(b.s)$. A discrimination tree stores the access words and discriminator suffixes learned for an SFA as per the following definition:

**Definition 4.** *Discrimination tree (adapted from [3]). A discrimination tree $\mathcal{T}$ is a tuple $(N, L, T)$ where $N$ is a set of nodes, $L \subseteq N$ is a set of leaves, and $T \subset N \times N \times \mathbb{B}$ is the transitions relation. Each leaf $l \in L$ is associated with a corresponding access word ($aw(l)$). Each internal node $i \in N \backslash L$ is associated with a discriminator suffix $d(i)$. For each element $(p, n, b) \in T$, $p$ is the parent node of $n$ and if $b = true$ (respectively $b = false$) we say that $n$ is the* accept *(respectively,* reject*) child of $p$.*

For a leaf $l \in L$ and inner node $n \in N \backslash L$, if $l$ is in the subtree of $n$ rooted in its accept child, then $\mathcal{M}(aw(l).d(n)) = true$. Similarly, if $l$ is in the reject subtree of $n$, $\mathcal{M}(aw(l).d(n)) = false$. In other words, the concatenation of $aw(l)$ with the discriminator suffix of any of its ancestor nodes is accepted iff $l$ is in the accept subtree of the ancestor node. For any two leaves $l_i, l_j \in L$ let $n_{i,j}$ be their lowest common ancestor in the DT. Then the discriminator suffix $d(n_{i,j})$ allows to discriminate the two states corresponding to $l_i$ and $l_j$ since $\mathcal{M}(aw(l_i).d(n_{i,j})) \neq \mathcal{M}(aw(l_j).d(n_{i,j}))$, with $aw(l_i).d(n_{i,j})$ being the accepted word if $l_i$ is in the accept subtree of $n_{i,j}$, or the rejected word otherwise.

**Learning algorithm.** We will here refer to the functioning of $MAT^*$ [3], although the main concepts apply to DT-based learning in general. To initialize the DT, the learner performs a membership query on the empty string $\epsilon$. The initial discrimination tree will be composed of two nodes: the root and a leaf node, both labeled with $\epsilon$. Depending on the outcome of the membership query, the leaf will be either the accept or the reject child of the root.

Given a word $w \in \Sigma^*$, to identify the state reached by parsing it according to the DT, the learner performs an operation called *sift*. Sift traverses the tree starting from its root $r$. For each internal node $n$ it visits, it executes the membership query $\mathcal{O}_m(w.d(n))$ to check whether $w$ concatenated with the discriminator suffix of $d$ is accepted by the target language. If it is accepted, sift continues visiting the accept child of $n$, and the reject child otherwise. If a leaf is reached, the learner concludes that parsing $w$ the target SFA reaches the state identified by the leaf's access word. If instead the child node sift should traverse next does not exist, a new leaf is created in its place with access word $w$. Membership queries of the form $a.\sigma$, where $a$ is an access string in the DT and $\sigma \in \Sigma$ are then issued to discover transitions of the SFA, possibly leading to the discovery of new states.

**Induced SFA.** A discrimination tree DT induces an SFA $\mathcal{M}_{DT} = (\mathcal{A}, Q, q_{init}, F, \Delta)$. In this paper, we assume $\mathcal{A}$ to be the Boolean algebra over the union of disjoint intervals over $\Sigma$. $Q$ is populated with one state $q_l$ for each leaf $l \in L$ of DT. The state $q_\epsilon$ is the initial state. If $\mathcal{O}_m(aw(l)) = true$, then $q_l \in F$ is a final state of $\mathcal{M}_{DT}$. To construct the transition relation $\Delta$, sifts of the form $aw(l).\sigma$ for $\sigma \in \Sigma$ are issued for the states $q_l$ and the concrete evidence for a transition between two states $q_i$ and $q_j$ is summarized into a consistent predicate of $\mathcal{A}$ using a partition function, as described for $\Lambda^*$.

**Counterexamples.** The equivalence query $\mathcal{O}_e(\mathcal{M}_{DT})$ will either confirm the learner identified the target language or produce a counterexample $c \in \Sigma^*$. As for $\Lambda^*$, the existence of $c$ implies that either a transition predicate is incorrect or that there should be a new state. To determine the cause of $c$, the first step is to identify the longest prefix $c[: i]$ before the behavior of the hypothesis SFA diverged from the target language. To localize the divergence point, the learner analyzes the prefixes $c[: i]$ for $i \in [0, len(c)]$. Let $a_i$ be the access string of the state of $\mathcal{M}_{DT}$ reached parsing $c[: i]$. If $\mathcal{O}_m(a_i.w[i :]) \neq \mathcal{O}_m(c)$, $i$ is the divergence point, which implies that the transition taken from $q_{a_i}$ is incorrect. Let $q_j$ be the state corresponding to the leaf reached when sifting $a_i.c[i :]$. The predicate guarding the transition between $q_{a_i}$ and $q_j$ is incorrect if $c[i]$ does not satisfy the corresponding transition predicate. This is possible because the partition function assigns the symbols in $\Sigma$ for which no concrete evidence is available to any of the outgoing transitions of $q_{a_i}$. In this case, the transition predicates should be recomputed to account for the new evidence from $c$. If instead $c[i]$ satisfies the transition predicate between $q_{a_i}$ and $q_j$, a new state should be added such that parsing $c[i]$ from $q_{a_i}$ reaches it. To add the new state, the leaf labeled with $a_i$ is replaced by a subtree composed of three nodes: an internal node with discriminator suffix $c[i :]$ having as children the leaf $a_i$ and a new leaf labeled by the access string $j.c[i]$, where $j$ is the access string of the state $q_j$ obtainened by sifting $a_i.c[i :]$. This procedure is called *split* (for more details, see, e.g., [3]). The updated DT will then be the base for the next learning iteration.

**Running example.** A DT corresponding to the running example introduced in Section 3 is shown below. While the specific structure of the learned DT depends on the order in which words are added to it, all the DT resulting from the learning process induce the same classification of the words $w \in \Sigma^*$, being them consistent representations of the same target language.

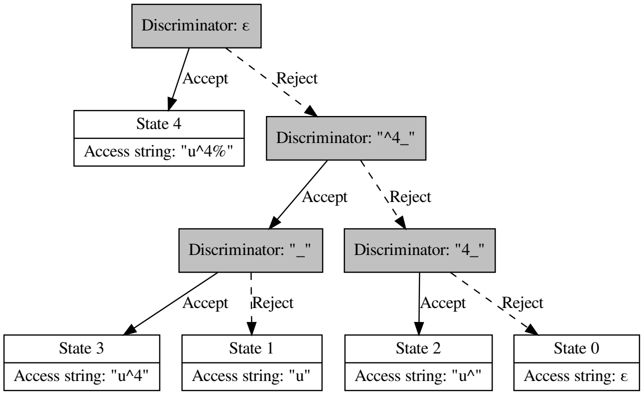

**Fig. 5.** Discrimination tree learned for the example of Section 3.

### 4 Active learning with concolic execution

The state-of-the-art active learning algorithms formalized in the previous sections are of limited use when trying to infer (an approximation of) the input language accepted by a program. Their main limitation is the reliance on a complete equivalence oracle, which is unavailable in this case.

In this section, we will propose several extensions of the $\Lambda^*$ and $MAT^*$ algorithms formalized in Sections 3.1 and 3.2 that make use of a concolic execution engine to 1) gather enhanced information from membership queries thanks to the path condition computed by the concolic engine, and 2) mitigate the lack of an equivalence oracle using the concolic engine to find counterexamples for a hypothesis. While it is usually unrealistic to assume a complete concolic execution of a large program (which would per se be sufficient to characterize the accepted input language), the ability of the concolic engine to execute each execution path only once brings significant benefits in our preliminary evaluation. Additionally, because the concolic engine can ask a constraints solver to produce inputs with a bounded length, it can be used to prove bounded equivalence between the learned input SFA and the target language. Finally, the availability of a partial symbolic execution tree and a constraint solver enables the definition of more effective types of membership queries.

#### 4.1 Concolic learning with symbolic observation tables

Given a program $\mathcal{P}$ its concolic execution on a word $w \in \Sigma^*$ produces a boolean outcome (accept/reject) and a path condition capturing the properties of $w$ that led to that outcome. In particular, we assume the path condition to be reducible to the constraint language defined in Section 2.4, i.e., the conjunction of interval predicates on the elements $w_i$ of $w$ and its length $len(w)$. Under this assumption, the path condition is directly translatable to a word $w_\Psi$ over the predicates $\Psi$ of the Boolean algebra $\mathcal{A}$ over the union of intervals over $\Sigma$. We will therefore refer to the path condition produced by the concolic execution of a word $w \in \Sigma^*$ with the $\Psi$-predicate as $w_\Psi$, where $len(w) = len(w_\Psi)$.

**Symbolic observation table.** The surjective relation between concrete words $w$ and their predicates $w_\Psi$ enables a straightforward extension of the observation table used for $\Lambda^*$, where the rows of the table can be indexed by words $w_\Psi \in \Psi^*$ instead of concrete words from $\Sigma^*$, i.e., $S \cup R \subset \Psi^*$. This allows for each row index to account for the entire equivalence class of words $w \in \Sigma^*$ that would follow the same execution path (these words will also have the same length). We describe as $[\![w_\Psi]\!]$ a concrete representative of the class $w_\Psi$. The set of suffixes $E \subset \Sigma^*$ will instead contain concrete elements of the alphabet.

**Membership queries.** Executing a membership query of the form $\mathcal{O}_m([\![w_\Psi]\!].\sigma)$, with $\sigma \in \Sigma$, will produce both the boolean outcome (accept/reject) and a word over $\Psi^*$ that can be added to $R$, if not already present. As a result, the transition predicates of the induced SFA can be obtained directly from the symbolic observation table, avoiding the need for a partition function to synthesize $\Psi$-predicates from the collected concrete evidence, as required in $\Lambda^*$. The transition relation is then completed by redirecting every $\sigma \in \Sigma$ that does not satisfy any of the discovered transition predicates to an artificial sink state. The induced SFA is then used as hypothesis for the next equivalence query.

**Equivalence queries.** Because a complete equivalence oracle for the target language is not available, we will use concolic execution to obtain a bounded equivalence oracle comparing the hypothesis SFA induced by the symbolic observation table with the program under analysis. To this end, we translate the hypothesis SFA into a function in the same programming language of the target program $\mathcal{P}$ that takes as argument a word $w$ and returns true (respectively, false) if the hypothesis SFA accepts (respectively, rejects) the word. We assume $\mathcal{P}$ to be wrapped into an analogous boolean function. We then write a program asserting that the result of the two functions is equal and use the concolic engine to find an input word that violate the assertion. If such word can be found, the counterexample is added to the symbolic observation table and the learner starts another iteration. If the concolic execution terminates without finding any assertion violation, it can be concluded that the hypothesis SFA represent the input language of $\mathcal{P}$. However, it is usually unrealistic to assume the termination of the concolic execution. Instead, we configure the solver to search for counterexamples up to a fixed length $n$. Assuming this input bounded concolic execution terminates without finding a counterexample, it can be concluded that the hypothesis is equivalent to $\mathcal{P}$'s input language for every word up to length $n$. Notably, this implies that if the target language is actually regular and the corresponding minimal automata has at most $n$ states, the hypothesis learned the entire language.

**Running example.** A symbolic observation table inducing the SFA for the example introduced in Section 3 is shown in Figure 6. The use of $\Psi$ predicates to index its rows significantly reduces the size of the table, since each row index accounts for a possibly large number of concrete elements of $\Sigma$.

| **T** | ϵ | _$1) | $1) | 1) | ) |
|---:|---|---|---|---|---|
| ϵ | - | + | - | - | - |
| 0-9; [-^; 0-9; :-\uffff | + | + | + | + | + |
| 0-9; [-^; 0-9 | - | + | + | - | + |
| 0-9; [-^ | - | + | - | + | - |
| 0-9 | - | + | + | - | - |
| a-z; a-z | - | + | + | - | - |
| _; ‘; {-\uffff | - | + | - | - | - |
| _; [-^ | - | + | - | + | - |
| 0-9; 0-9 | - | + | + | - | - |
| a-z; :-@ | - | + | - | + | - |
| _; [-^; [-^ | - | + | - | - | - |
| A-Z; A-Z | - | + | + | - | - |
| \u0000-/ | - | + | - | - | - |
| [-^ | - | + | - | - | - |
| A-Z; _; {-\uffff; 0-9 | - | + | + | - | + |
| 0-9; :-@; 0-9; 0-9 | - | + | + | - | - |
| A-Z; \u0000-/; :-@ | - | + | - | - | - |
| 0-9; [-^; \u0000-/ | - | + | - | - | - |
| A-Z; :-@ | - | + | - | + | - |
| a-z | - | + | + | - | - |
| A-Z; _ | - | + | + | - | - |
| _; ‘ | - | + | - | + | - |
| _ | - | + | + | - | - |
| 0-9; :-@; 0-9 | - | + | + | - | + |
| A-Z; :-@; A-Z | - | + | + | - | - |
| ‘ | - | + | - | - | - |
| A-Z; ‘; 0-9; :-\uffff | + | + | + | + | + |
| :-@ | - | + | - | - | - |
| 0-9; {-\uffff | - | + | - | + | - |
| {-\uffff | - | + | - | - | - |
| A-Z; \u0000-/ | - | + | - | + | - |
| A-Z; _; {-\uffff; 0-9; \u0000-/ | + | + | + | + | + |
| _; [-^; a-z | - | + | + | - | - |
| A-Z | - | + | + | - | - |
| A-Z; ‘; 0-9; :-\uffff; \u0000-\uffff | + | + | + | + | + |
| A-Z; :-@; ‘ | - | + | - | - | - |
| a-z; :-@; _ | - | + | + | - | - |

**Fig. 6.** Symbolic observation table for the example of Section 3.

#### 4.2 Concolic learning with a symbolic membership oracle

In the previous section, we used the concolic engine to extract the path conditions corresponding to the execution of membership queries produced by the learner. This enabled reducing the number of queries – each query gathering information about a set of words instead of a single one – and keeping the observation table more compact. In this section, we introduce an oracle that answers a new class of *symbolic membership queries (SMQs)* using the constraint solving capabilities of the concolic engine to directly compute predicates characterizing all the accepted words of the form $p.\sigma.s$, where $p, s \in \Sigma^*$ and $\sigma \in \Sigma$. This oracle will enable a more efficient learning algorithm based on an extension of the discrimination tree data structure.

**Definition 5.** *Symbolic Membership Oracle* ($\mathcal{O}_s$)*. Given a Boolean algebra* $\mathcal{A}$ *with predicate set* $\Psi$*, a* symbolic membership oracle $\mathcal{O}_s : \Sigma^* \times \Sigma^* \to \Psi$ *takes as input a pair* $(p, s)$ *and returns a predicate* $\psi \in \Psi$ *such that for a symbol* $\sigma \in \Sigma$*,*

*the target program accepts $p.\sigma.s$ iff $\sigma \models \psi$. $p$ and $s$ are called* prefix *and* suffix*, respectively.*

An SMQ query can be solved by issuing a membership query for each $\sigma \in \Sigma$. However, this operation would be costly for large alphabets, such as unicode. On the other hand, the concolic execution of $w = p.\sigma.s$ for a concrete symbol $\sigma$ returns via the path condition the entire set of symbols that wold follow the same execution path, in turn leading to the same execution outcome. A constraint solver can then be used to generate a new concrete input outside of such set, which is guaranteed to cover a new execution path. This procedure is summarized in Algorithm 1, where we use $pathCondition[\sigma]$ to represent the projection of the path condition on the element of the input string $w = p.\sigma.s$ corresponding to the position of $\sigma$.

**Input:** SMQ $Q = (p, \psi, s)$; $concolic : \Sigma^* \rightarrow$(accepted, pathCondition)
**Result:** $\psi$ such that $\forall \sigma \in \Sigma : p.\sigma.s$ is accepted iff $\sigma \models \psi$

```
ψ ← ⊥;
unknown ← Σ;
while unknown ≠ ∅ do
    σ ← pickElementFrom(unknown);
    accepted, pathCondition ← concolic(p.σ.s);
    if accepted then
        ψ ← ψ ∨ pathCondition[σ];
    end
    unknown ← unknown ∧ ¬ pathCondition[σ];
end
return ψ;
```

**Algorithm 1:** Answering SMQ queries.

**Learning transition predicates with $\mathcal{O}_s$.** Consider the learning algorithm using discrimination tree introduced in Section 3.2, $MAT^*$. After each iteration, the discrimination tree DT contains in its leaves all the discovered states (identified by the respective access words) and organized according to their discrimination suffixes (labeling the internal nodes of DT). To construct the transition relation of the induced SFA, the algorithm executes for each leaf $l$ and $\sigma \in \Sigma$ a sift operation to determine the state reached when parsing $aw(l).\sigma$. Each such sift operation requires as many membership query as the depth of the reached state to be determined. Therefore, the number of sift operations needed to construct the complete transition relation is proportional to the number of states times the size of the input alphabet, with each sift operation issuing a number of membership queries proportional to the depth of DT.

Using the symbolic membership oracle, we can instead define a procedure that traversing DT directly synthesize the transition predicate between a source state $q_s$ and a destination state $q_t$ of the induced SFA. This procedure is formalized in Algorithm 2.

**Input:** $DT = (N, L, T)$; $\mathcal{O}_s : \Sigma^* \rightarrow \psi$; source state $q_s$; target state $q_t$
**Result:** The transition predicate $\pi$ between $q_s$ and $q_t$
$n \leftarrow$ root of DT;
$\pi \leftarrow \top$;
**while** $n \in N \backslash L$ **do**
  $\psi \leftarrow \mathcal{O}_s(aw(q_s), d(n))$;
  **if** $q_t$ *in the accept subtree of* $n$ **then**
    $\pi \leftarrow \pi \wedge \psi$;
    $n \leftarrow acceptChild(n)$;
  **else**
    $\pi \leftarrow \pi \wedge \neg\psi$;
    $n \leftarrow rejectChild(n)$;
  **end**
**end**
**return** $\pi$;

**Algorithm 2:** Learning transition predicates with $\mathcal{O}_s$.

Algorithm 2 allows to construct the induced SFA by computing for each ordered pair of leaves of DT the transition predicate of the corresponding transition. This results in the direct construction of the complete transition relation of the induced SFA. In practice, the implementation of Algorithm 2 can be improved by observing that $\pi_i$ computed in the $i$-th iteration of the loop is by construction a subset of $\pi_{i-1}$. The symbolic membership oracle $\mathcal{O}_s$ can make use of this observation to limit the search procedure for the construction of the predicate $psi$ during the $i$-th iteration to only symbols that satisfy $\pi_{i-1}$, significantly improving its efficiency. Finally, for the same reason, the loop in Algorithm 2 can terminate as soon as $\pi = \bot$, which indicates that no transition exists between the source and destination states.

*Example.* Referring to the discrimination tree in Figure 5 for the example introduced in Section 3, assume we want to learn transition predicate from State 2 to State 3. Initially, $\pi_0 = \top$. The access string of State 2 is "u^". The suffix of the root node is $\epsilon$. Invoking the symbolic membership oracle, we obtain $\psi = \mathcal{O}_s(\text{"u\^{}"}, \epsilon) = \bot$ (no string of length 3 are accepted by the target language). Because State 3 is in the reject subtree, $\pi_1 = \pi_0 \wedge \neg\bot = \top$ and the execution moves to the internal node labeled with the discriminator suffix "^4_". The corresponding SMQ query returns $\psi = \mathcal{O}_s(\text{"u\^{}"}, \text{"\^{}4\_"}) = \{0 \dots 9, A \dots Z, \_, a \dots z\}$. Because State 3 is in the accept subtree of the current node, $\pi_2 = \pi_1 \wedge \psi = \{0 \dots 9, A \dots Z, \_, a \dots z\}$ and the execution moves to the internal node with discriminator prefix "_", where $pi_3 = [0 \dots 9]$ is finally computed as the transition predicate from State 2 to State 3.

**Decorated discrimination tree.** For every leaf $l$ and internal node $n$ of a discrimination tree DT, Algorithm 2 issues a SMQ query $(aw(l), d(n))$. The corresponding intermediate value of the transition predicate $\pi$ is intersected with the result of the SMQ query or its negation depending on whether $l$ is in the accept or the reject subtree of $n$. Notably, the addition of a newly discovered state to DT does not change the relative positioning of a leaf $l$ with respect to an internal node $n$, i.e., if $l$ is initially in the accept (respectively, reject) subtree of $n$, it will remain in that subtree also after a new state is added. This observation implies that the results of the SMQ queries performed through Algorithm 2 remain valid between different executions of the algorithm. Therefore, when a new state is discovered and added to the discrimination tree via the *split* operation defined in Section 3.2, only the membership queries involving the new internal node and the new leaf added by *split* would require an actual execution of the symbolic membership oracle.

To enable the reuse of previous SMQ queries issued through Algorithm 2, we decorate the DT adding to each node a map from the set of leaves $L$ to the value of $\pi$ computed when traversing the node. We refer to this map as *predicate map*. Every predicate in the root node map is $\top$, as this is the initial value of $\pi$ in Algorithm 2. The maps in the children nodes are then computed as follows. Let $n$ be a parent node and $n_a$, $n_r$ its accept and reject children respectively, $m$ be a leaf of DT, and $\pi_n^m$, $\pi_{n_a}^m$, $\pi_{n_r}^m$ the predicates for $m$ stored in $n$, $n_a$, and $n_r$, respectively. Then $\pi_{n_a}^m = \mathcal{O}_s(aw(m), d(n)) \wedge \pi_n^m$ and $\pi_{n_r} = \neg\mathcal{O}_s(aw(m), d(n)) \wedge \pi_n^m$. Proceeding recursively a leaf $l$ will be decorated with a predicate map assigning to each leaf $l_i$ in DT the predicate of the transition going from $q_{l_i}$ to $q_l$.

Figure 7 shows the decorated version of the discrimination tree of Figure 5, constructed for the same example language introduced in Section 3.

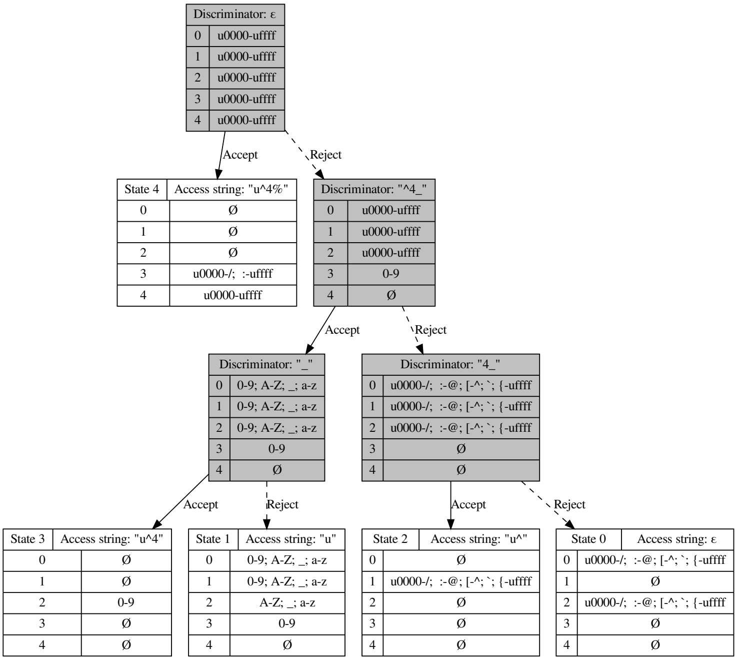

**Fig. 7.** Decorated version of the discrimination tree in Figure 5.

**Induced SFA and number of equivalence queries.** Notice that, by construction, for every node $n$ with accept child $n_a$ and reject child $n_r$, if $\pi^l_n$, $\pi^l_{n_a}$, and $\pi^l_{n_r}$ are the predicates the three nodes associate with a leaf $l$, $\pi^l_{n_a} \vee \pi^l_{n_r} = \pi^l_n$ and $\pi^l_{n_a} \wedge \pi^l_{n_r} = \bot$. As a consequence, after all the maps decorating a node in the discrimination tree are completed, the predicates in the leaves represent the complete transition relation of the induced SFA. Further more, the maps grows monotonically through the learning process, with entries computed in previous iterations remaining valid throughout the entire process. Practically, after each split operation resulting from the counterexample of an equivalence query (see Section 3.2), we traverse the discrimination tree and incrementally update all the predicate maps to include information about transitions to the new leaf, as well as populating the maps of the new internal node and new leaf added by the split.

Differently from the original algorithm $MAT^*$ described in Section 3.2, a counterexample for the induced SFA corresponding to a decorated discrimination tree can only be returned if a new state has been discovered. This bound the number of equivalence query to be at most equal to the minimum number of states needed to represent the target language as an SFA. In our settings, a complete equivalence oracle is not available for the target program $\mathcal{P}$. Equivalence queries are instead solved using a (input bounded) concolic execution that compares the hypothesis SFA (induced by the discrimination tree) with the original program. Because this execution is computationally expensive, reducing the number of necessary equivalence queries has a significant impact on the execution time (at the cost of keeping in memory the node predicate maps).

## 5 Experimental evaluation

### 5.1 Experimental Setup

In this section we evaluate a prototype implementation of our contributions, built upon SVPAlib [9] (the symbolic automata and alphabet theory library used by MAT*) and Coastal [12], a concolic execution engine for Java bytecode. in Section 5.2 we consider our approach of using symbolic observations tables from Section 4.1 (referred to as SymLearn in the following presentation) and in Section 5.3 we evaluate the use of the symbolic membership queries from Section 4.2 (referred to as MAT*++). All the experiments have been executed on a server equipped with an AMD EPYC 7401P 24-Core CPU and 440Gb of memory. Coastal was configured to use at most 3 threads using its default generational exploration strategy [12] to find counterexamples for equivalence queries.

The experiments in this section are based on regular expressions taken from the AutomatArk [8] benchmark suite. To ensure a uniform difficulty distribution among the experiments, the regular expressions were converted to their automaton representation, sorted by the number of states, and 200 target automata selected by a stratified sampling (maximum number of states in an automaton is 637 and average 33; maximum number of transitions 2, 495 and average 96). Each automaton is then translated into a Java program accepting the same language and compiled. The program analysis is performed on the resulting bytecode.

In the first experiment, we demonstrate the increase in query efficiency we achieve, by comparing the number of queries, using a complete oracle that can answer equivalence queries in a negligible amount of time. In this idealised setup the learner halted when the correct automaton was identified, relying on the fact that the oracle can confirm the correctness of the hypothesis. Although this setup does not represent a realistic scenario, it allowed us to reliably evaluate the number of queries of each type that are required to converge to the correct automaton, and to measure the computational requirements of the learning algorithm in isolation.

In the second experiment, we demonstrate the use of a concolic engine as a symbolic oracle, and measure the impact on the execution time of the algorithms. Providing a meaningful evaluation of the cost of the equivalence queries is difficult, as it is essentially a software verification problem over arbitrary Java code, and in principle an equivalence query could never terminate. Instead, a complete concolic analysis of each parser is performed, without using the perfect oracle for any type of query, and enforcing a timeout of ten minutes for each analysis, after which the learner yielded it's latest hypothesis. The correctness of that hypothesis is then confirmed by comparison to the known target automata. Note also that we use an input string length limit of 30 for the words to be parsed during concolic execution.

#### 5.2 Learning with symbolic observation tables

**Evaluating the algorithm with a perfect oracle.** We learn 78% of the target languages within the ten minute timeout using a perfect oracle for equivalence. We see a 54% reduction in the total number of membership queries, and a 88% reduction in the total number of equivalence queries over MAT* (see Table 1).

**Table 1.** Number of queries and execution time with perfect oracle.

| Algorithm | Membership queries | Equivalence queries | Execution time (s) |
|---|---|---|---|
| MAT* | 1,545,255 | 25,802 | 38.60 |
| SymLearn | 720,658 | 3,124 | 1321.70 |

The SymLearn approach requires the path conditions to be stored in the observation table, even when using a perfect oracle for equivalence. In order to achieve this, concrete counter examples from the perfect oracle are resolved to path conditions via the concolic engine. The slower execution time can be attributed to the infrastructure overhead present in our implementation, and the speed of the concolic engine when performing these resolutions.

**Evaluating the algorithm with the concolic oracle.** We now replace the perfect equivalence oracle with a concolic execution engine, as described in Section 4.1. We learn 30% of the target automata within the ten minute timeout. The execution time is orders of magnitude slower when compared MAT*, and in our implementation 99% of the learner's execution time is spent running symbolic equivalence queries. While the increase in bandwidth due to the path conditions returned for each query does result in a significant reduction of queries overall, the execution time of the SymLearn approach is orders of magnitude slower than MAT*, partly because SymLearn requires the actual (concolic) execution of the program implementation, instead of performing queries on an SFA representation of the regular expression. There are however a number of optimizations that can be made to improve the performance (some of which will be discussed in the following section).

#### 5.3 Learning with symbolic membership queries

Under the assumption that the language to be learned is regular, and that the equivalence check will eventually find a counterexample if there exists one, our active learning approach guarantees that eventually the correct hypothesis will be generated. The experimental evaluation was therefore aimed at understanding what is achievable in a realistic setting, with constrained time, and how our methodology improves the outcome.

**Table 2.** Number of queries and execution time with perfect oracle.

| | Membership queries | SMQ | Equivalence queries | Learner execution time |
|---|---|---|---|---|
| MAT* | 3,517,474 | – | 47,374 | 137.51 s |
| MAT*++ | 42,075 | 81,401 | 1,913 | 1.33 s |

Table 2 shows the total number of queries[3] necessary to learn the correct automaton over the 200 test cases, along with the CPU time used by the learner process alone, without considering the time required to answer the queries.

The decrease in the CPU time required by the learner process can be explained by the reduction in the number of counterexamples that the learner has to process (recall that in MAT*++, a counterexample can be caused only by a missing state in the hypothesis, while in MAT* it can be also be due to an incorrect transition predicate). To understand the balance between the benefit due to the sharp reduction in the number of membership and equivalence queries, and the cost due to the introduction of the symbolic queries, the next section will evaluate the cost of answering each type of query without the help of a perfect oracle.

**Evaluating the impact of SMQ.** First, observe that the impact of membership queries are negligible since it is simply a check to see if an input is accepted. However, measuring the complexity of the symbolic membership queries (SMQs) is crucial to assess the effectiveness of our approach. Answering a SMQ requires the concolic execution of the program under analysis potentially multiple times, and requires processing each resulting path condition to collect the information needed to refine the answer. In this experiment we measured the time and the

[3] Note that all 200 automata are included in Table 2 whereas only the results for the subset that finished before the timeout was shown in Table 1.

number of concolic executions required to answer all the SMQs of table 2. The total time required to answer the queries was 4,408s, with an average of 54.15 ms per query. The number of concolic executions per query was between 1 and 31, with an average of 3.45. Since the concolic execution requires the program under analysis to be instrumented and a symbolic state to be maintained, it is orders of magnitude slower than a standard concrete execution.

**Evaluating the impact of equivalence queries.** Each equivalence query is answered in the same way as in the SymLearn approach (see Section 4.1), by doing a concolic execution of the hypothesis and the program being analyzed on the same symbolic input to see if they give a different result; if so, we have a counter-example, otherwise we simply know none could be found before the timeout or within the input string length of 30. As a further optimization, we also maintained two automata *knownAccept* and *knownReject* that were the union of the automata translation of the path conditions of all the previously explored accepted and rejected inputs respectively.

In this experiment 1,207 equivalence queries were issued, and on average it took 56.92s to answer a query. 573 answers were generated in negligible time using the *knownAccept* and *knownReject* automata (demonstrating the usefulness of this optimization), 93 cases Coastal could not find a counter-example (within the input size limit), 107 the timeout occurred and in the rest a counter-example was found by Coastal. In 152 cases the correct automaton was learned (76%), and interestingly in 62 of these cases Coastal timed-out (but the current hypothesis at the time was in fact correct). In 3 of the cases Coastal finished exploring the complete state-space up to the 30 input before the timeout, but the correct automaton was not learned. This happened because a counter-example requiring more than 30 input characters exist.

**Discussion of the results.** The benefit of the symbolic membership queries is clear: it reduces the number of equivalence queries by 96%, and the latter is by far the most expensive step in active learning without a perfect oracle. Furthermore, simple engineering optimizations, for example a caching scheme for the accepted and rejected path conditions, can have a significant impact on the execution time.

### 6 Related work

The problem of learning input grammars has been tackled using a variety of techniques, and with various specific goals in mind.

#### 6.1 Active learning

The active learning algorithms most closely related to our are $\Lambda^*$ [11] and MAT* [3], which have been extensively discussed in Section 3.

Argyros et al. [4] used Angluin-style active learning of symbolic automata for the analysis of finite state string sanitizers and filters. Being focused on security, their goal was not to learn exactly the filter under analysis, but to verify that it filters every potentially dangerous string. In the proposed approach each equivalence query is approximated with a single membership query, which is a string that is not filtered by the current hypothesis, but belongs to the given language of "dangerous" strings. If no such string exists, the filter is considered successfully validated. If the string exists but is successfully filtered it provides a counterexample with which the hypothesis is refined, otherwise a vulnerability in the filter has been found. This equivalence approximation is incomplete, but greatly simplifies the problem, considering the complexity of equivalence queries.

Multiple other approaches use different active learning techniques not based on L* that, compared to our solution, provide less theoretical guarantees and often rely on a corpus of valid inputs. Glade [6] generates a context free grammar starting from a set of seed inputs that the learner attempts to generalize, using a membership oracle to check whether the generalization is correct. No other information is derived from the execution of the program under analysis, and therefore the set of seed inputs is of crucial importance. Reinam [29] further extends Glade by using symbolic execution to automatically generate the seed inputs, and adding a second probabilistic grammar refinement phase in which reinforcement learning is used to select the generalization rules to be applied.

The approach proposed by Höschele et al. [19] uses a corpus of valid inputs and applies generalizations that are verified with a membership oracle. Dynamic taint analysis is used to track the flow of the various fragments of the input during the execution, extracting additional information that aids in the creation of the hypothesis and generates meaningful non-terminal symbol names. A similar approach is used by Gopinath et al. [16], with the addition of automatic generation of the initial corpus.

#### 6.2 Passive learning

Godefroid et al. [15] use recurrent neural networks and a corpus of sample inputs to create a generative model of the input language. This approach does not learn any information from the system under test, so the sample corpus is important.

A completely different approach is used by Lin et al. [24] to tackle a related problem: reconstructing the syntax tree of arbitrary inputs. The technique is based on the analysis of an execution trace generated by an instrumented version of the program under analysis. This approach relies on the knowledge of the internal mechanisms used by different types of parsers to generate the syntax tree.

Tupni [7] is a tool to reverse engineer input formats and protocols. Starting from one or more seed inputs, it analyzes the parser execution trace together with data flow information generated using taint analysis, identifies the structure of the input format (how the data is segmented in fields of various types, aggregated in records, and some constraints that must be satisfied), and generates a context free grammar.

### 7 Conclusions

Most established active learning algorithms for (symbolic) finite state automata assume the availability of a minimal adequate teacher, which includes a complete equivalence oracle to produce counterexample disproving an incorrect hypothesis of the learner. This assumption is unrealistic when learning the input grammar of a program from its implementation, as such a complete oracle would need to automatically check the equivalence of the hypothesis with arbitrary software code. In this paper, we explored how the use of a concolic execution engine can improve the information efficiency of membership queries, provide a partial input-bounded oracle to check the equivalence of an hypothesis against a program, and enable the definition of a new class of symbolic membership queries that allow the learner inferring the transition predicates of a symbolic finite state automata representation of the target input language more efficiently.

Preliminary experiments with the Autmatark [8] benchmark showed that our implementations of SymLearn and MAT*++ achieve a significant reduction (up to 96%) in the number of queries required to actively learn the input language of a program in the form of a symbolic finite state automaton. Despite bounding the total execution time to 10 minutes, using the concolic execution engine as partial equivalence oracle, MAT*++ managed to learn the correct input language in 76% of the cases.

This results demonstrate the suitability of concolic execution as enabling tool for the definition of active learning algorithms for the input grammar of a program. However, our current solutions learn the input grammar in the form of a symbolic finite state automaton. This implies that only an approximation of non-regular input languages can be constructed. Such approximation can at best match the input language up to a finite length, but would fail in recognizing more sophisticated language features that may require, for example, a context free representation. Investigating how the learning strategies based on concolic execution we explored in this paper can generalize to more expressive language models is envisioned as a future direction for this research, as well as the use of the inferred input languages to support parsers validation and grammar-based fuzzing.
